# Progress and challenges in adaptive robotics

**DOI:** 10.3389/frobt.2022.1020462

**Published:** 2022-10-24

**Authors:** Stefano Nolfi

**Affiliations:** Institute of Cognitive Sciences and Technologies, National Research Council, Roma, Italy

**Keywords:** adaptive robotics, reinforcement learning, evolutionary robotics, machine learning, embodiment

## Introduction

Adaptive robotics achieved tremendous progress during the last few years (see Nolfi (2021) for an introduction and review). The term adaptive robotics refers to methods which permit the design of robots capable of developing their skills autonomously through an evolutionary and/or learning process. It focuses on approaches requiring minimal human intervention in which the behavior displayed by the robots and the control rules producing such behavior are discovered by an adaptive process automatically on the basis of a reward or fitness function which rates how well the robot is doing. It focuses on end-to-end learning, i.e. on systems which receive as input directly the state of robot’s sensors and determine directly the state of the robot’s actuators, without involving any type of hand-designed pre-processing. Finally, it focuses on model-free methods, i.e. on systems which do not have an internal model of the environment, or in which the internal model is acquired automatically during the adaptation process. In this article I will review the major advances and the research challenges.

## Advances

The first examples of adaptive robots date back to many years ago (Mahadevan & Connell, 1992; Nolfi et al., 1994). These works, however, were restricted to rather simple robots and tasks and to robots characterized by low-dimensional observation and action spaces. Successful applications of adaptive methods to complex problems were achieved only recently. Those include robots capable of displaying dexterous manipulation (Gu et al., 2017; Andrychowicz et al., 2018) and bipedal walking (Salimans et al., 2017; Yu, Turk & Liu, 2018).

A first major advance was achieved through the development of more powerful adaptive algorithms. The introduction of deep learning techniques, such as modern stochastic optimizers and regularization methods (see Arulkumaran et al., 2017 for a review), permitted to scale-up reinforcement learning methods to problems that were previously intractable. Moreover, the development of modern evolutionary strategies, which uses a form of finite difference method to estimate the gradient of the expected fitness [see Pagliuca, Milano & Nolfi [2020]; Salimans et al., 2007; Pagliuca, Milano & Nolfi (2020)], permitted to scale-up evolutionary methods to problems involving high-dimensional observation and action spaces.

A second major advance concerned the reality-gap problem. Adaptive approaches generally require long training processes. Carrying the training in hardware is feasible but expensive (see for example Levine et al., 2017). Moreover, it usually requires designing special devices to calculate the reward and to periodically reset the environment. Carrying the training in simulation is much more convenient and permits to speed up the process through the usage of parallel computation. The development of domain randomization methods permit to obtain robots which can cross the reality gap, i.e. which can keep working properly once moved from simulation to the real world. Domain randomization, originally proposed by Jakobi et al. (1995), is realized by randomly sampling different simulation parameters during the training of the robot. The parameters subjected to variations can include dynamic parameters of the robot and of the environment (Peng et al., 2018; Tan et al., 2018) and visual and rendering parameters such as texture and lighting (Sadeghi & Levine, 2017; Tobit et al., 2017).

The usage of simulation also permits to improve and speed-up learning by exploiting the information contained in the ground-truth state of the robot and of the environment which is available in simulation and which cannot be accessed in hardware. Such information can be used to compute the reward and/or can be provided in input to the critic which is used to estimate the expected reward during the training process (for an example, see Andrychowicz et al., 2018).

Finally, a third major advance regards the development of methods and techniques which improve the exploration capacity of the adaptive process thus reducing the risk to incur in stagnation or local minima.

Intrinsic motivation (Badia et al., 2010; Schmidhuber, 2010) achieves this objective by rewarding the robots also for displaying new behaviors and/or experiencing new observations. The rationale behind the approach is that the new behaviors acquired in this way can be later reused to produce functional behaviors. Similarly, novel observations can promote the development of new functional behaviors afforded by them.

Curriculum learning manipulates the learning experiences of the robot to facilitate the adaptation process and to challenge the weakness of the adaptive robot. This is realized by varying the characteristics of the evaluation episodes so as to expose the robot to conditions which are difficult but not too difficult and which challenge the weaknesses of the adapting robot (see for example Milano & Nolfi, 2021). Alternatively, it is realized by storing the previous learning experiences in a replay buffer and by choosing the samples on the basis of some measure of usefulness. The priority can be given to the samples which generate the highest learning progress (Schaul et al., 2015), the samples with the highest complexity (Ren et al., 2018), or the samples which are less common (Cohn et al., 2016).

Competitive co-evolution (Lan, Chen & Eiben, 2019; Simione & Nolfi, 2021) or self-play (Bansal at al., 2018) expose the learning robots to environmental conditions which become progressively more difficult and challenging. This is realized by training a robot for the ability to defeat a competitor and by concurrently training the competitor for the ability to defeat the robot. This form of adversarial learning can produce an open-ended process in which the abilities of the robot and the complexity of the task keep increasing in an unbounded manner.

Finally, experience replay (Andrychowicz et al., 2017) permits generating positive training data. This is obtained by transforming the training data leading to failure with respect to a given objective to training data leading to success with respect to a different objective, i.e. the objective which corresponds to the actual outcome of the robot’s behavior. Generating positive training data is particularly useful in problems in which the probability to receive positive rewards is initially low.

The aspects discussed above are still actively investigated as the topics reviewed in the next Section. The difference lies in the fact that the former already produced consolidated results.

## Promising research directions

In this section, I will briefly illustrate promising research directions that may enable substantial further progress in the field.

A first research line concerns the usage of modular architectures supporting knowledge re-use. The importance of knowledge re-use is demonstrated by the efficacy of convolutional neural networks which are commonly used for vision processing. Such efficacy is largely due to the fact that the same connection weights are used for processing different sub-sections of the image. Modular architecture of different kinds suited to process different types of information might provide similar advantages. The architecture proposed by Huang et al. (2020) to control the joints of multi-segments robots (see also Wang et al., 2018) represents an interesting proposal of this kind. The model includes neural modules that have identical connection weights. Each module controls a corresponding joint and receives inputs from the local sensors only. The differentiation of the activity of the joints is obtained through messages passed between neighboring modules which propagate to distant modules. As shown by the authors a single modular policy can successfully generate locomotion behaviors for robots with varying morphologies and can generalize to new morphologies not encountered during training such as creatures with extra legs.

A second important research line concerns the developments of methods supporting the development of multiple behaviors and behavior re-use. Current research focuses on the development of a single skill from scratch. Such skills might involve lower-level skills which are instrumental for achieving the corresponding function. On the other hand, the behavioral repertoire which is functional to the achievement of a single goal is limited. We should find methods enabling the robots to progressively expand their behavioral repertoire during the adaptation process in an open-ended manner. This also involves the synthesis of systems with multi-level and multi-scale organizations in which the lower level skills are combined and re-used to produce higher level skills (Nolfi, 2021).

Finally, a third important research line concerns world models, i.e. the possibility to design agents capable of acquiring a model of the world and of their interaction with the world and to use it to maximize their expected reward (Ha & Schmidhuber, 2018a and, 2018b; Hafner et al., 2018, 2020). Such world models can incorporate the large amount of information possessed by humans and animals which is usually indicated with the term common sense (Le Cunn, 2022). Examples of common sense knowledge are the fact that the world is tridimensional, the fact that the world includes objects of different kinds, the fact that objects preserve their properties and move smoothly *etc.* Common sense knowledge can be acquired conveniently through a form of latent or self-supervised learning. The challenge is thus to design robots capable of acquiring a model of the world through self-supervised learning, capable of exploiting the common sense knowledge acquired to improve their adaptive capability, and eventually capable of using their world model to reason and plan mentally without necessarily interacting with the external environment.

The latent learning process which can be used to acquire the world model can be realized by training the robot’s neural network to capture the mutual dependencies between its inputs, e.g. by training the robot to predict future observation and rewards on the basis of the previous observations and on the basis of the action that the robot is going to perform. The idea to use neural networks and self-supervision to learn models for control is not new and has been proposed originally in the 90s by Jordan & Rumelhart (1992). The interest in the idea renewed after the proposal of new methods which overcome the problem caused by the fact that the world is only partially predictable by predicting a representation of the state of the world instead of directly the state of the world. This is realized by learning concurrently how to represent the world and how to predict the next representation on the basis of the previous representations. Moreover, it is realized by choosing representations which maximize both the information preserved in the representation and the predictability of future representations (Le Cunn, 2022).

The world model can be used in two different modalities which correspond to the “System 1” and “System 2” components described by Daniel Kahneman (2011), see Le Cunn (2022). In the first case the policy produces the action directly on the basis of the observation and on the basis of the state of the world model which anticipates the future state of the world. In the second case, the agent reasons and plans by using the world model. More specifically, it proposes an initial sequence of actions, uses the world model to compute future states of the world and reward, propose better action sequences, and finally execute the action sequence. The action sequence to be executed can be obtained by using a form of dynamic programming (Bertsekas, 2019) or by identifying the best action directly through a gradient-based method.

Although the first realizations of the idea (Ha & Schmidhuber, 2018a and, 2018b; Hafner et al., 2018, 2020) are promising, several aspects still represent open challenges. A first challenge concerns the identification of methods ensuring that the data experienced are sufficiently rich and varied to acquire an effective world model. A second challenge concerns the identification of methods ensuring that the learning process does not become unstable. Finally, a third challenge concerns the identification of how the System-2 component can be implemented in detail and can be integrated with the System-1 component.

## Conclusion

Developing intelligent robots capable of acquiring their skills autonomously in interaction with the environment is one of the most ambitious objectives of science. The challenges which are still open are substantial but appear feasible in light of the progresses achieved in the last years.
